# Edge Effects on Simultaneous Reconstructions of Flame Temperature and Soot Volume Fraction Profiles by a CCD Camera

**DOI:** 10.3390/s22218199

**Published:** 2022-10-26

**Authors:** Jie Li, Hongtao Li, Chen Chen, Guannan Liu, Yaoyao Ying, Tianjiao Li, Dong Liu

**Affiliations:** 1 MIIT Key Laboratory of Thermal Control of Electronic Equipment, School of Energy and Power Engineering, Nanjing University of Science and Technology, Nanjing 210094, China; 2 Advanced Combustion Laboratory, School of Energy and Power Engineering, Nanjing University of Science and Technology, Nanjing 210094, China; 3 Department of Electronic Engineering, School of Electronic and Optical Engineering, Nanjing University of Science and Technology, Nanjing 210094, China

**Keywords:** edge effect, simultaneous reconstruction, flame temperature profile, soot volume fraction, reconstruction boundary

## Abstract

In this paper, the influence of the edge effect on the simultaneous reconstruction of axisymmetric flame temperature and soot volume fraction profiles by a single CCD camera was investigated in detail. The reconstruction accuracy of the flame temperature profile and soot volume fraction was insensitive to the measurement error of the coefficient matrix. When the signal to ratio (*SNR*) of the measurement system for both the radiation intensity and coefficient matrix was as low as 46 dB, the reconstruction accuracy for both temperature and soot volume fraction was acceptable and was more influenced by the radiation intensity measurement error. The reconstruction of the flame temperature and soot volume fraction was greatly influenced by the edge effect. When the flame edge with weak radiation signals was ignored during the reconstruction, the relative reconstruction error for the temperature and soot volume fraction increased from the flame center to the edge, and reached an unacceptable value at the reconstruction boundary, especially for the soot volume fraction. The flame image boundary could be chosen as the unified reconstruction boundary to reconstruct the two-dimensional distribution of the temperature and soot volume fraction with satisfactory accuracy. The low soot volume fraction could influence the reconstruction accuracy for both the temperature and soot concentration in non-sooting regions. Moreover, there was no obvious regularity between the reconstruction accuracy of the temperature and soot volume fraction and the extension of the reconstruction boundary.

## 1. Introduction

The rapid development of the economy is accompanied by the huge consumption of energy. The world’s primary energy and carbon emissions both fell by the most since World War II in 2020 during the COVID-19 pandemic, but fossil fuels such as coal, oil, and natural gas still play a dominant role in the world’s energy consumption structure, and countries, as well as organizations around the world, have correspondingly made commitments to achieve carbon neutrality and carbon peaking [1]. Restrictions on the utilization of fossil fuels are not only due to their non-renewability but also related to the toxic and harmful pollutants generated during the combustion of them, such as nitrogen oxides, carbon oxides, unburned hydrocarbons and soot, of which soot is the most important combustion pollutant. It is well-known that soot can cause glaciers to melt, haze, and other environmentally unfriendly phenomena on the one hand, on the other hand, it can penetrate deep into the respiratory system and circulatory system, causing harm to human health [2,3,4,5]. Flame temperature is one of the most intuitive and important parameters to reflect the combustion state. Meanwhile, the temperature and soot generation as well as annihilation during combustion are not independent, and they affect each other. Therefore, the accurate measurement of the flame temperature and soot property is crucial.

Compared with traditional contact thermometry, optical diagnostic methods have been widely used due to their non-invasiveness and reliable accuracy. Among the optical diagnostic methods, the laser-based measurement methods are more preferred by researchers because of the satisfactory accuracy, but the sophisticated and expensive equipment as well as the complex calibration process limit its application to almost laboratory-scale flame [6,7,8,9,10]. As a high-temperature radiation source, the radiation information contained in the flame itself can reflect the characteristics and changes of the many parametric fields of the flame. With the continuous development of imaging technology in recent years, a variety of cameras with excellent performance have been utilized to capture the radiation signals of the flame, and with the subsequent image processing techniques as well as the algorithms for solving inverse problems, the spatial distribution of the expected flame temperature and soot concentration is available with satisfactory precision.

Since laboratory flames are easier to manipulate, a large number of researchers tend to quantify their temperature and soot concentration to verify the reliability of reconstruction models. Liu et al. [9] simultaneously utilized laser-induced fluorescence (LIF), laser-induced incandescence (LII), and two-color pyrometry combined with Abel transformation to study the effect of flame temperature on soot formation and evolution in the partially premixed and diffusion flames of a diesel surrogate under different dilution gases atmosphere. Huang et al. [11] proposed a reconstruction model based on a stereoscopic image system which was validated by the numerical reconstruction under different noise levels as well as hypotheses of soot complex refraction index and the subsequent experimental measurements of the flame temperature and soot volume fraction on both axisymmetric and asymmetric flames. Das et al. [12] investigated the influence of the addition of macromolecular hydrocarbons on combustion by inversing the temperature and soot volume fraction with the Gaussian basis set expansion Abel transform method. Ai et al. [13] presented a reconstruction model for axisymmetric flames in combination with the three-color emission method to retrieve the temperature and soot volume fraction of both single-peaked flame and double-peaked flame, and the numerical results reflected the superiority compared with the traditional two-color thermometry. Subsequently, the reconstruction model was optimized by Yan et al. [14,15,16] to reconstruct the temperature and soot loading of an ethylene diffusion flame under different combustion conditions considering the spectral response efficiency of the CCD camera. Recently, Li et al. [17] coupled the TR-GSVD algorithm into the reconstruction model to inverse the temperature and soot concentration of the partially premixed flames and inverse diffusion flames. Kuhn et al. [18] put forward a new method of absolute radiation intensity calibration that imaged the incandescent light of a SiC filament with known emissivity in the flame, and with two DSLR cameras, the flame temperature and soot volume fraction were obtainable. Currently, Serwin and Karatas [19] proposed a calibration method that did not need to rely on standard radiation sources to calibrate the absolute radiation intensity in the process of the tomography inversion of the flame temperature and soot concentration. This method relied on the LOSA method to measure the extinction coefficient at first, and the global calibration factor between the soot volume fraction, the radiation source term as well as the camera exposure time was obtained subsequently under the condition of ignoring the scattering so that the flame temperature and soot concentration could be reconstructed simultaneously.

The combustion diagnostic method based on flame self-emission imaging was toilless to implement with merely imaging devices, hence, numerous research has been manipulated on industrial combustion. Zhou et al. [20] applied the improved Tikhonov regularization method to the tomographic inversion of the radiation energy image obtained from the CCD cameras arranged at the four corners of the furnace, and reconstructed the convergence values of temperature, absorption coefficient, and wall emissivity by the alternate update of three parameters. Lou et al. [21] and Cheng et al. [22] optimized the reconstruction model and further carried out the reconstruction of temperature and nonuniform radiation properties of soot in the coal-fired furnace and gas-fired pilot tubular furnace, respectively. Liu et al. [23,24] presented an inverse radiation model for the concurrent estimation of the temperature and radiative properties, which introduced the backward Monte Carlo method to trace the radiative transfer of the flame radiation energy captured by four CCD cameras mounted staggered to each other on the four sides of the two-dimensional rectangular area.

With the gradual diversification of combustion systems and the continuous development of imaging technology, the application of flame diagnosis methods based on image processing is becoming more and more diversified. Guo et al. [25] developed a Python-based toolbox to automatically scan the flame image captured by a single digital camera and carry out the subsequent imaging-processing steps for calculating the flame temperature and soot volume fraction in the flames of condensed phased fuels. Liu et al. [26,27,28,29,30,31] established a detailed and complete reconstruction model for temperature and particle concentration estimation in both axisymmetric and asymmetric complicated combustion systems consisting of soot and nanoparticles. The reliability and the precision of the model were verified numerically, but the radiation energy collected by ordinary cameras only included the wavelength information of the camera’s *RGB* channels, which meant that the spectral resolution was low to a certain extent. Snelling et al. [32] systematically elaborated the realization process of the multispectral reconstruction of the flame temperature and soot concentration, which combined the spectrometer and CCD sensors, and the correction for self-absorption was presented. The reconstruction model was utilized in pressurized combustion diagnosis, subsequently [33,34,35]. Liu et al. [36] presented a reconstruction method to obtain temperature and soot volume fraction based on the decomposition of the emissivity with an integrative hyperspectral device and both the simulation and experimental results were satisfactory. The transfer process of radiation energy from the flame to the imaging device is complex and variable, and the propagation of the radiation energy mentioned in the above research was assumed or simplified, while the light field cameras, which could record the intensity and direction of the light propagation, provided a novel idea for the reconstruction of the temperature and soot parametric field. Wen et al. [37] developed a hybrid Landweber method and a sequential quadratic programming algorithm to handle the images captured by the light-field camera, and the relative reconstruction error for the three-dimensional temperature and absorption coefficient was within 5% and 12%, respectively. George et al. [38] investigated the influence of four different algorithms on the reconstruction of temperature and soot volume fraction based on light-field imaging technology, and the results were consistent with those measured by thermocouples and extinction method with a relatively low spatial resolution.

The research mentioned above reflected the development of a flame parametric field reconstruction based on image processing in many fields; however, the flame itself as a dispersion medium and the radiation energy in the entire area of the flame was inhomogeneous. The radiation energy near the flame edge gradually faded away, which was not discussed in detail in the former research. Li et al. [39] proposed an iterative multiwavelength method to reconstruct the temperature and soot volume fraction in an absorbing, emitting flame area with a meshing of rectangles; the results showed that the relative reconstruction error near the flame edge was also large, but a further detailed discussion was not carried out. As a consequence, the edge effects on the reconstruction of the flame temperature and soot volume fraction distributions were investigated in this paper. Firstly, the impact of the coefficient matrix—the calculation of which was closely related to the choice of the flame reconstruction edge—on the reconstruction of the flame cross-section temperature and soot volume fraction was studied. Secondly, the reconstruction performance was further evaluated under the situation that the flame edge with a weak radiation signal was ignored during the reconstruction. Finally, a method for diminishing the edge effect was proposed and the reliability of the reconstruction model was validated with an assumed two-dimensional distribution of the flame temperature and soot volume fraction.

## 2. Reconstruction Model

### 2.1. The Numerical Model of the Direct Problem

**Figure 1:**
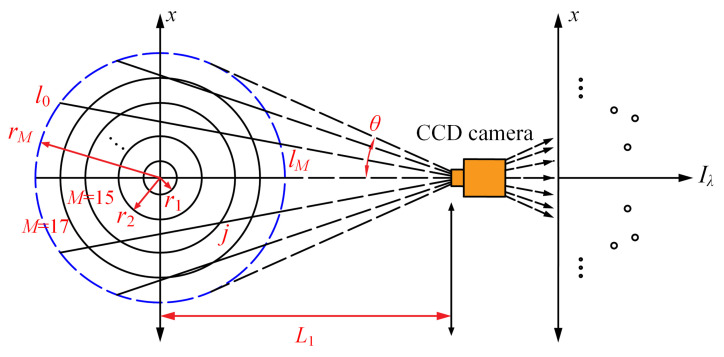
The reconstruction model considering the edge effect.

The system diagram of the reconstruction model considering the edge effect is shown in Figure 1, which was the common reconstruction model as elaborated in refs. [26,27,28,29,30,40]. The cross-section of an axisymmetric laboratory flame was divided into *M* equal-spaced concentric circles, and the flame temperature and soot volume fraction in the elemental area enclosed by each circle was assumed to be uniform, namely, the quantity variance of both temperature and soot concentration at the same radius was ignored. A CCD camera was installed on the axis of the flame cross-section to capture the flame radiation intensity. With the hypothesis that the flame radiation from the gas component was neglected, as can be seen in Figure 1, the flame radiation was thought to result from the accumulation of radiation emitted by soot within each ring and the quantified radiation bisected the field angle at which the flame fell within the camera’s field of view.

The condensed soot in the flame was assumed to be the sparse particle system such that the radiation characteristic of the system was considered to be the superposition of radiation properties of individual particles, including the assumption that the size of soot fell within the Rayleigh scattering range [41]. With the hypotheses above, the emitted radiation intensity for ray *j* could be written according to the radiative transfer equation as follows [41].

(1) $$I_{\lambda }(j)=\int_{l_{0}\left(j\right)}^{l_{m}\left(j\right)}\kappa_{\lambda }{(l)I}_{b,\;\lambda }(l)\exp[-\int_{l(j)}^{l_{m}\left(j\right)}\kappa_{\lambda }(l{}^{\prime })dl{}^{\prime }]dl\;\;\approx \;\int_{l_{0}\left(j\right)}^{l_{m}(j)}\kappa_{\lambda }{(l)I}_{b,\;\lambda }(l)dl$$

where $\kappa_{\lambda }\left(l\right)$ and $I_{b,\;\lambda }($*l*) represent the spectral absorption coefficient and the spectral radiation intensity of the blackbody along the radiation transfer path, respectively; $\exp\;[-\int_{l(j)}^{l_{m}\left(j\right)}\kappa_{\lambda }\left(l{}^{\prime })dl{}^{\prime }\right]$ is the description of the self-absorption term and with the optically thin assumption, it can be neglected.

Based on the discretized flame cross-section and flame radiation in Figure 1, the radiation intensity expressed by the integral form in Equation (1) could be rewritten in discretized form as follows.

(2) $$I_{\lambda }(j)=\int_{l_{0}\left(j\right)}^{l_{m}(j)}\kappa_{\lambda }{(l)I}_{b,\;\lambda }(l)dl=\overset{M}{\underset{m=1}{\sum }}\kappa_{\lambda }{(m)I}_{b,\;\lambda }{(m)l}_{j}\left(m\right)=\overset{M}{\underset{m=1}{\sum }}H_{\lambda }{(m)l}_{j}\left(m\right)$$

where $\kappa_{\lambda }(m)$ and $I_{b,\;\lambda }\left(m\right)$ represent the local spectral absorption coefficient and the local blackbody spectral radiative intensity in the *m*th ring, respectively, and the product of them form the local radiation source term $H_{\lambda }\left(m\right)$; $l_{j}\left(m\right)$ represents the path length that ray *j* transferred through in the *m*th ring; *M* represents the number of the flame elemental ring; *N* represents the number of radiation lines.

The relation between the emitted radiation intensity and the desired flame parameters, namely, flame temperature and soot volume fraction were established through Wien’s law and Rayleigh approximation [41], as shown in Equations (3) and (4), respectively.

(3) $$I_{b,\;\lambda }(m)=\frac{c_{1}}{{\pi \lambda }^{5}\exp[\frac{c_{2}}{\lambda T(m)}]}$$

(4) $$\kappa_{\lambda }(m)=\frac{{36\pi n}_{\lambda }k_{\lambda }}{{{(n}_{\lambda^{2}}-{k_{\lambda }}^{2}+2)}^{2}+4{n_{\lambda }}^{2}{k_{\lambda }}^{2}}\frac{f_{v}\left(m\right)}{\lambda }$$

where *T*(*m*) represents the flame temperature in the *m*th ring; $c_{1}$ is the first radiation constant; $c_{2}$ is the second radiation constant; $n_{\lambda }$ and $k_{\lambda }$ represent the real and imaginary parts of the soot complex refraction index, respectively, and in this paper, they are calculated as follows [42].

(5) $$N_{\lambda }=1.811+0.1263\ln\lambda +{0.027\ln}^{2}\lambda +{0.0417\ln}^{3}\lambda$$

(6) $$K_{\lambda }=0.5821+0.1213\ln\lambda +{0.2309\ln}^{2}\lambda -{0.01\ln}^{3}\lambda$$

For all the flame radiation rays, the radiation transfer equations constitute the system of linear equations, which can be written in matrix form as follows.

(7) $$I_{\lambda }=L\cdot H_{\lambda }$$

where $I_{\lambda }$ represents the spectral emissive radiation intensity vector of the flame ($I_{\lambda }\in R^{N}$); **L** is the coefficient matrix calculated according to the intersection of radiation ray and flame elemental ring ($L\;\in {\;R}^{N\times M}$); $H_{\lambda }$ represents the spectral radiation source vector ($H_{\lambda }\in R^{M}$).

For the direct problem, the coefficient matrix **L** and the radiation source term $H_{\lambda }$ were assumed to be known and that the emissive radiation intensity vector $I_{\lambda }$ was available, which was implemented by the presumptive distribution of flame temperature and soot concentration universally. When the influence of the edge effect on the reconstruction of flame temperature and soot concentration was considered, in the direct problem, the outer boundary (*M* = 17) of the reconstruction model was chosen as the reconstruction boundary for the flame cross-section.

### 2.2. The Solving Model of the Inverse Problem

For the inverse problem, the emissive radiation intensity vector $I_{\lambda }$ was known after being calculated from the direct problem, and the coefficient matrix **L** as a geometric quantity was also known, so the goal of the inverse problem was to obtain the accurate distribution of radiation source term $H_{\lambda }$. When investigating the influence of the edge effect on the reconstruction accuracy for flame temperature and soot concentration, in the inverse problem, the radiation intensity near the flame edge might be so weak that it caused obvious reconstruction error; therefore, the area near the flame edge was ignored for the reconstruction. In this paper, the situations wherein the 17th elemental ring and both 16th and 17th elemental rings were ignored were investigated. For instance, as shown in Figure 1, when the 17th elemental ring was ignored, the area enclosed by the 17th circle and 16th circle was ignored, and the emissive radiation intensity for ray j was still the total radiation intensity that passed through all 17 rings, but the coefficient matrix was calculated considering the cases that passed through 16 rings, and the path length that transferred through 17th ring was ignored. Consequently, the matrix equation for the inverse problem was shown as follows.

(8) $$I_{\lambda }{}^{\prime }=L{}^{\prime }\cdot H_{\lambda }{}^{\prime }$$

where the superscript symbol represents the subset of the corresponding matrix or vector.

Both Equations (7) and (8) were ill-posed equations with many zero elements in the coefficient matrix, so the least-squares QR factorization (LSQR) algorithm [43,44] was utilized to acquire the optimal solution of the equation. In this paper, the center wavelengths of the *R* and *G* channel of the camera (700 nm and 530 nm) were used during the reconstruction procedure, and after the dichromatic radiation source term was acquired, the flame temperature and soot volume fraction could be calculated with colorimetry as shown below.

(9) $$T(m)=\frac{c_{2}(\frac{1}{\lambda_{2}}-\frac{1}{\lambda_{1}})}{\ln\frac{H_{\lambda_{1}}\left(m\right)}{H_{\lambda_{2}}\left(m\right)}-\ln C+5\ln\frac{\lambda_{1}}{\lambda_{2}}}$$

(10) $$C=\frac{\frac{n_{\lambda_{1}}k_{\lambda_{1}}}{{{{(n}_{\lambda_{1}}}^{2}-{k_{\lambda_{1}}}^{2}+2)}^{2}+4{n_{\lambda_{1}}}^{2}{k_{\lambda_{1}}}^{2}}\frac{1}{\lambda_{1}}}{\frac{n_{\lambda_{2}}k_{\lambda_{2}}}{{{{(n}_{\lambda_{2}}}^{2}-{k_{\lambda_{2}}}^{2}+2)}^{2}+4{n_{\lambda_{2}}}^{2}{k_{\lambda_{2}}}^{2}}\frac{1}{\lambda_{2}}}$$

(11) $$f_{v}(m)=\frac{H_{\lambda_{1}}\left(m\right)}{{36\pi I}_{{b,\;\lambda }_{1}}\left(m)(\mathrm{CC}\right)}$$

(12) $$CC=\frac{n\lambda_{1}k_{\lambda_{1}}}{{{{(n}_{\lambda_{1}}}^{2}-{k_{\lambda_{1}}}^{2}+2)}^{2}+{{4n}_{\lambda_{1}}}^{2}{k_{\lambda_{1}}}^{2}}\frac{1}{\lambda_{1}}$$

## 3. Results and Discussion

### 3.1. Effect of Measurement Error

**Figure 2:**
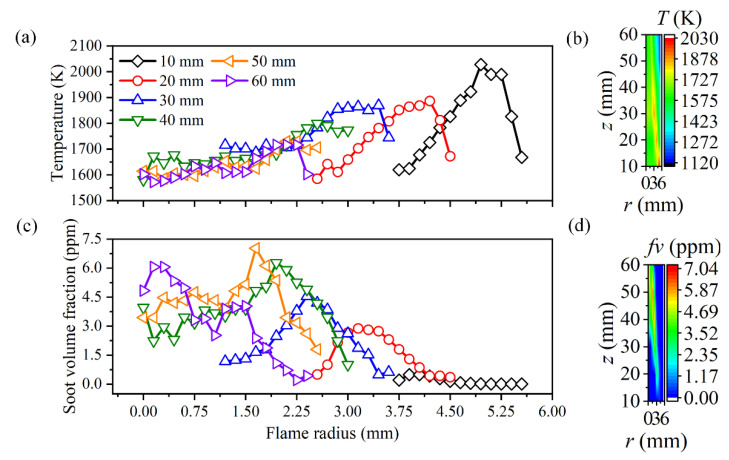
The exact data of temperature and soot volume fraction [45]. (**a**) The main cross-section flame temperature in the sooting regions; (**b**) The 2D distribution of flame temperature; (**c**) The main cross-section soot volume fraction in the sooting regions; (**d**) The 2D distribution of soot volume fraction.

As discussed above, the essence to validate the reliability of the reconstruction model was to compare the solution in the inverse problem and the presupposed distribution of the flame temperature and soot volume fraction in the direct problem. In this paper, the assumed distribution of the flame temperature and soot volume fraction is displayed in Figure 2, which was extracted from the line-of-sight measurement of a typical coaxial diffusion flame [45]. It should be noted that in some parts of the flame, such as in the center area of the lower part of the flame, there was no soot produced or the soot volume fraction was too weak to be detected due to the limitation of the line-of-sight method based on flame self-emission information in ref. [45]. Therefore, in these regions, the soot volume fraction was assumed to be an extremely low value (0.001 ppm), and they were called non-sooting regions below. In other regions, namely, the regions with a considerable flame temperature and soot volume fraction, as shown in Figure 2a,c, they were called sooting regions in the subsequent discussion. Due to the flame thermal radiation, the flame temperature in the non-sooting regions could still be maintained at a relatively high value, so the flame temperature in non-sooting regions was assumed according to its distribution in sooting regions. Additionally, the totals of the 2D distributions of the flame temperature and soot volume fraction are shown in Figure 2b,d, respectively.

In practice, the measurement error is inescapable during the measurement of the flame emissive radiation intensity. Meanwhile, as it can be seen in Figure 1, as a geometric quantity, the calculation of the coefficient matrix was relevant to the radius of the flame and the distance from the flame cross-section center to the CCD camera, as well as to the numbers of the flame elemental rings and radiation lines. The determination of the flame radius was relevant to the choice of the flame edge, which might cause a measurement error for the coefficient matrix and therefore a disturbance might be introduced in the matrix equation. Consequently, the stochastic errors of a normal distribution ξ with the zero mean value μ and mean square error σ as well as a normal distribution η with the zero mean value μ and mean square error σ were introduced to the radiation intensity and coefficient matrix, respectively, as shown in Equations (13) and (14).

(13) $$I_{\lambda ,\;\mathrm{CCD}}={(\mu +\sigma \xi )I}_{\lambda ,\;\mathrm{id}}{+I}_{\lambda ,\;\mathrm{id}}$$

(14) $$L_{\mathrm{calc}}=(\mu +\sigma \eta {)L}_{\mathrm{id}\;}{+L}_{\mathrm{id}}$$

where $I_{\lambda ,\;\mathrm{CCD}}$ represents the measured radiation intensity; $I_{\lambda ,\;\mathrm{id}}$ represents the accurate radiation intensity without noise; $L_{\mathrm{calc}}$ represents the coefficient matrix with measurement noise; and $L_{\mathrm{id}}$ represents the accurate coefficient matrix.

The logarithmic decibel scale was introduced to correlate the signal-to-ratio (*SNR*) of the system and the stochastic error [11].

(15) $$SNR=20\log10[\frac{1}{{\left(\frac{1}{N}\sum_{i=1}^{N}\sigma^{2}\zeta_{i}^{2}\right)}^{2}}]$$

To better evaluate the reconstruction precision of the reconstruction model, the relative reconstruction errors for both temperature and soot volume fraction were calculated as follows.

(16) $$E_{T,\;\mathrm{rec}}(m)=100\frac{{|T}_{\mathrm{rec}}{(m)-T}_{\mathrm{id}}\left(m)\right|}{T_{\mathrm{id}}\left(m\right)}$$

(17) $$E_{f_{v,\;\mathrm{rec}}}(m)=100\frac{{|f}_{v,\;\mathrm{rec}}{(m)-f}_{v,\;\mathrm{id}}\left(m)\right|}{f_{v,\;\mathrm{id}}\left(m\right)}$$

**Figure 3:**
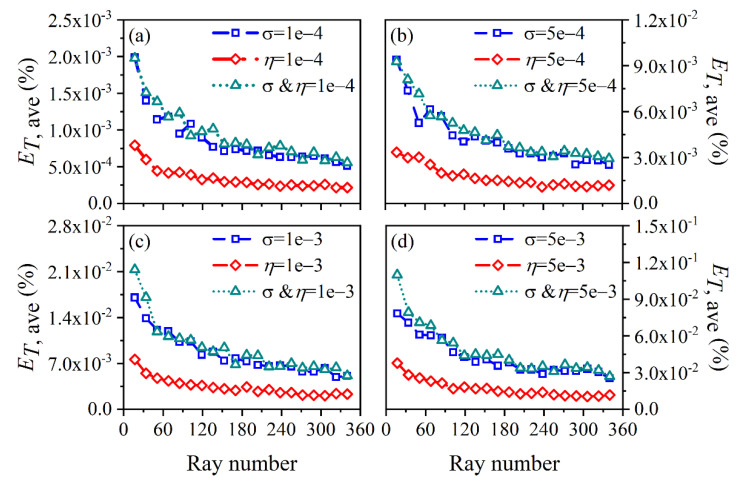
The average relative reconstruction error for temperature under different ray numbers considering the measurement error of the radiation intensity and coefficient matrix. (**a**) *SNR* = 80 dB; (**b**) *SNR* = 65 dB; (**c**) *SNR* = 60 dB; (**d**) *SNR* = 46 dB.

**Figure 4:**
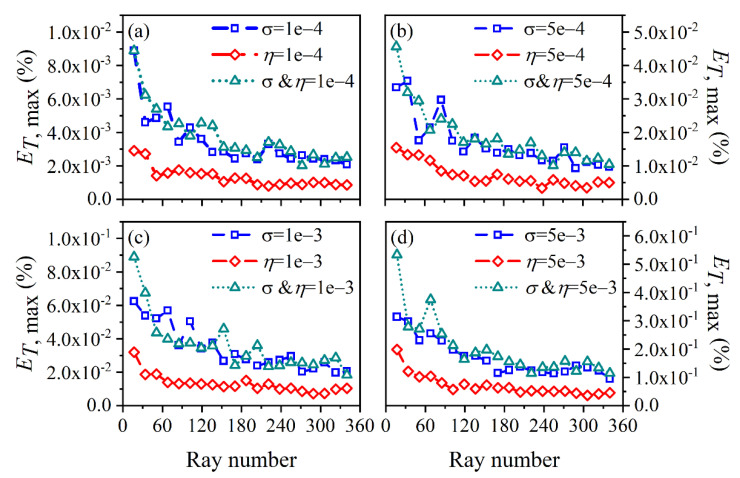
The maximum relative reconstruction error for temperature under different ray numbers considering the measurement error of the radiation intensity and coefficient matrix. (**a**) *SNR* = 80 dB; (**b**) *SNR* = 65 dB; (**c**) *SNR* = 60 dB; (**d**) *SNR* = 46 dB.

**Figure 5:**
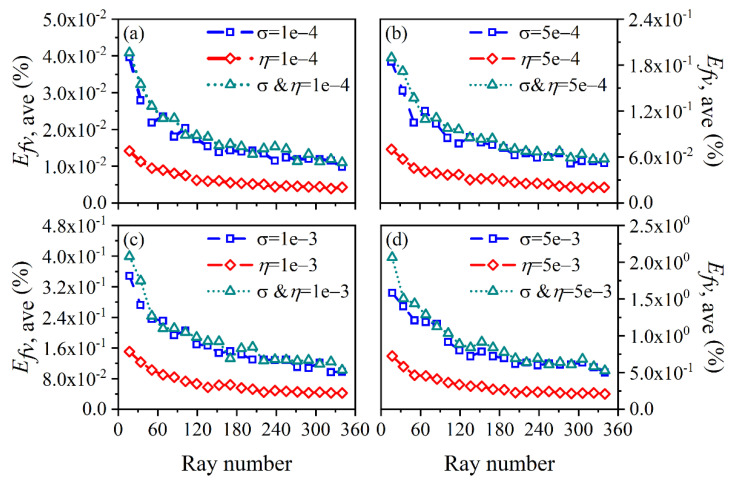
The average relative reconstruction error for soot volume fraction under different ray numbers considering the measurement error of the radiation intensity and coefficient matrix. (**a**) *SNR* = 80 dB; (**b**) *SNR* = 65 dB; (**c**) *SNR* = 60 dB; (**d**) *SNR* = 46 dB.

**Figure 6:**
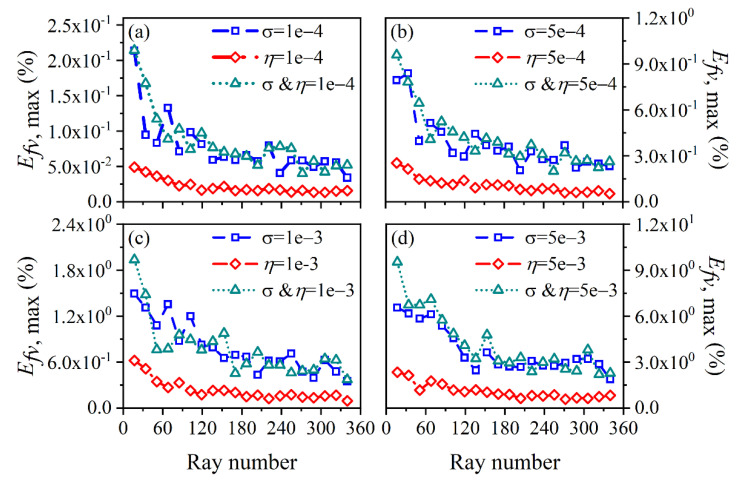
The maximum relative reconstruction error for soot volume fraction under different ray numbers considering the measurement error of the radiation intensity and coefficient matrix. (**a**) *SNR* = 80 dB; (**b**) *SNR* = 65 dB; (**c**) *SNR* = 60 dB; (**d**) *SNR* = 46 dB.

The average and maximum relative reconstruction error of temperature are shown in Figure 3 and Figure 4, respectively, while the average and maximum relative reconstruction errors for soot volume fraction are shown in Figure 5 and Figure 6, respectively. It was generally found that in either the flame temperature or the soot volume fraction, both the average and maximum relative reconstruction error increased with the decrease of the *SNR* of the system, and meanwhile, as the ray number increased, they decreased obviously first and then flattened out gradually. Compared to the maximum relative reconstruction error, the monotonicity between the average relative reconstruction error and the ray number was more apparent, especially for the flame temperature. Moreover, the relative reconstruction error of the flame temperature was significantly lower than that of the soot volume fraction.

In comparison to the measurement error of the radiation intensity, the measurement error of the coefficient matrix had much less influence on the reconstruction accuracy for both the flame temperature and soot volume fraction. In addition, when the same level of measurement noise existed in the radiation intensity and coefficient matrix, the measurement error of the radiation intensity played a major role in the reconstruction accuracy. The reason for the above rules was that whether the measurement noise was introduced in the radiation intensity or the coefficient matrix, the essence was in providing some disturbance to the reconstruction equation, so the relation between the reconstruction accuracy and the *SNR* of the system as well as the ray number showed a similar trend. However, the order of the magnitude of radiation intensity was much larger than the coefficient matrix; the measurement noise of which could exert a greater impact on the reconstruction precision. In the mass, the reconstruction precision was satisfactory such that when the *SNR* of the system was as low as 46 dB, the maximum of the average relative reconstruction error for temperature and soot volume fraction was 0.11% and 2.06%, respectively, while the maximum of the maximum relative reconstruction error for the temperature and soot volume fraction was 0.53% and 9.53%, respectively.

### 3.2. Influence of Edge Effect

As discussed above, the reconstruction accuracy for the flame temperature and soot volume fraction was insensitive to the measurement error of the coefficient matrix, but this conclusion was based on a certain coefficient matrix with some artificially introduced noise, while the calculation of the coefficient matrix was relevant to the choice of the reconstruction boundary, which was challenging to determine due to the dispersivity of the flame itself and the faint radiation signal near the flame edge. The idea of verifying the influence of the edge effect on the reconstruction accuracy of the flame temperature and soot volume fraction is mentioned in Section 2.

**Figure 7:**
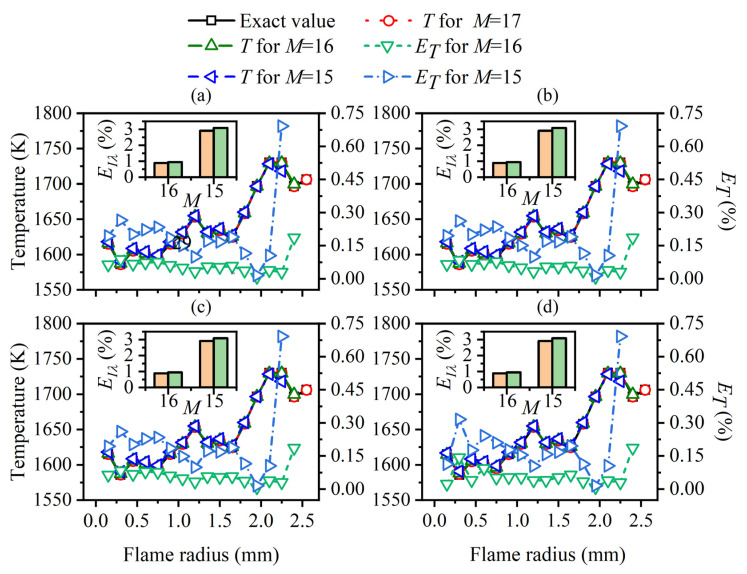
The reconstructed flame temperature and corresponding relative reconstruction error as well as the radiation intensity loss ratio under different cases of *SNR* considering the edge effect. (**a**) *SNR* = 80 dB; (**b**) *SNR* = 65 dB; (**c**) *SNR* = 60 dB; (**d**) *SNR* = 46 dB.

**Figure 8:**
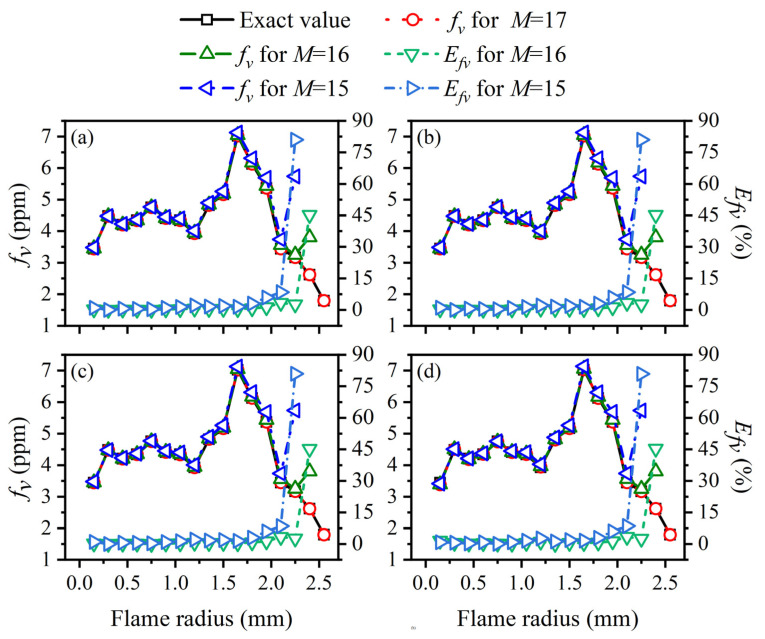
The reconstructed soot volume fraction and corresponding relative reconstruction error under different cases of *SNR* considering the edge effect. (**a**) *SNR* = 80 dB; (**b**) *SNR* = 65 dB; (**c**) *SNR* = 60 dB; (**d**) *SNR* = 46 dB.

The reconstructed flame temperature and soot volume fraction as well as the corresponding relative reconstruction error under different cases of *SNR* considering the edge effect are shown in Figure 7 and Figure 8, respectively, and the proportion of the emissive radiation intensity of the neglected region to the total emissive radiation intensity is also displayed in Figure 7.

As can be seen in Figure 7, when the 17th ring was neglected, the radiation intensity loss ratio was approximately 1% regardless of the *SNR* of the system, and when the 16th and 17th rings were both neglected, the radiation intensity loss ratio was about 3%. Meanwhile, as the flame radius increased, the relative reconstruction error of the flame temperature increased a lot near the reconstruction boundary, which was also insensitive to the *SNR* of the system. In addition, the more neglected the region, the larger the relative reconstruction error. Though the reconstruction accuracy of the temperature was worse near the reconstruction boundary compared to other regions, it was still in an acceptable range (less than 1%), while the relative reconstruction error of the soot volume fraction was much worse near the reconstruction boundary, with a maximum of 45.21% and 80.97% when the 17th ring was neglected and both the 16th and 17th ring were neglected, respectively.

**Figure 9:**
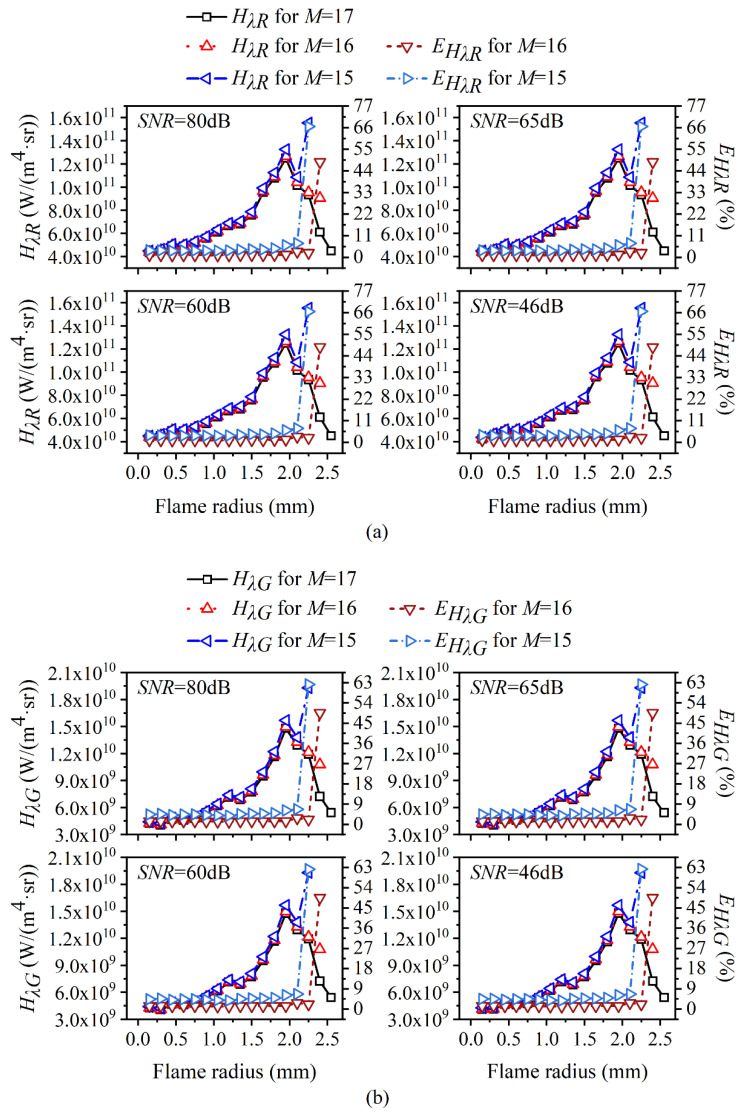
The influence of edge effect on the reconstruction of (**a**) *H_λR_*; (**b**) *H_λG_* under different cases of *SNR*.

**Figure 10:**
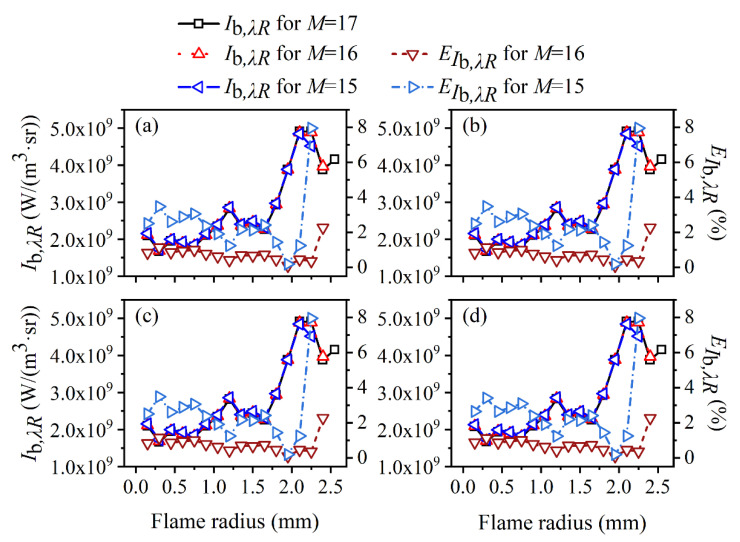
The influence of edge effect on local blackbody radiation intensity using red wavelength under different cases of *SNR*. (**a**) *SNR* = 80 dB; (**b**) *SNR* = 65 dB; (**c**) *SNR* = 60 dB; (**d**) *SNR* = 46 dB.

Considering that the calculation of the flame temperature depended on the ratio of the radiation source term under two different wavelengths, while the soot volume fraction was relevant to the ratio of the chromatic radiation source term to the blackbody radiation intensity, the reconstructed radiation source term considering the edge effect under two wavelengths is shown in Figure 9, and the calculated blackbody radiation intensity using red wavelength is shown in Figure 10. It can be seen from Figure 9 that the relative reconstruction error for the radiation source term augmented as the flame radius increased and reached the unacceptable maximum at the reconstruction boundary. Meanwhile, the relative reconstruction error of the radiation source term under two wavelengths was pretty close regardless of the edge effect, which explained why the relative reconstruction error of temperature was insensitive to the edge effect. Compared to the radiation source term, the relative reconstruction error of the blackbody radiation intensity under red wavelength, as shown in Figure 10, exhibited a similar trend with the augmentation of the flame radius, but the value was significantly lower so that the soot volume fraction was reconstructed with a large error.

As can be seen in Figure 1, when the area near the flame edge was neglected, the radiation rays that passed through the elemental rings in that area were also neglected. Additionally, the radiation ray near the neglected region passed through fewer flame elemental rings at the same time, which meant that the radiation intensity for the same radiation ray was the same in both the direct problem and inverse problem, but the crossing length was shorter in the inverse problem. This was equivalent to measuring energy attenuation with a shorter propagation path. From the matrix equation itself, the unknown number in the reconstruction equation for the area near the flame edge decreased, and some equations of many variables even became monadic equations. By contrast, the reconstruction equations for the area near the flame center had more variables, so the decrease in the crossing length had little influence on the equations as well as the relative reconstruction error of the flame temperature and soot volume fraction in the central area.

### 3.3. Two-Dimensional Parametric Field Reconstruction under Different Factors

As discussed above, the reconstruction accuracy of the cross-section flame temperature and soot volume fraction was insensitive to the measurement error of the coefficient matrix, while the improper choice of the reconstruction boundary to neglect the area with feeble radiation intensity could cause a large reconstruction error near the flame edge, especially for soot volume fraction. Considering that the flame and surrounding background with a weak radiation signal were both imaged into a two-dimensional rectangular area, and the procedure to calculate the coefficient matrix of all the flame cross-sections was cumbersome, thereby, the image boundary was proposed to be the unified reconstruction boundary in the following discussion to access the reconstruction performance of the whole two-dimensional flame area. The assumed two-dimensional flame temperature and soot volume fraction as well as the radial distribution of them in sooting regions are displayed in Figure 2. The maximum flame radius was 5.55 mm, and it was chosen as the unified reconstruction boundary first. The number of flame elemental rings was 38, and the number of radiation rays passing through the half-flame section was doubled from 38.

**Figure 11:**
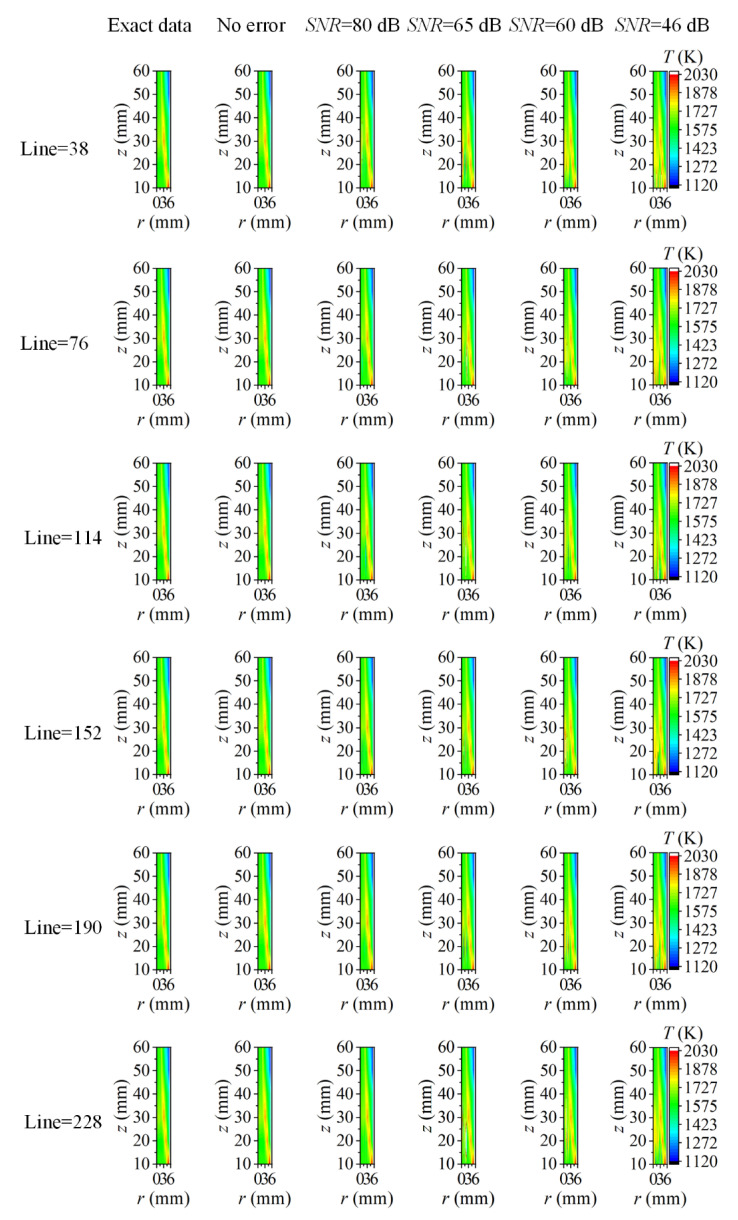
The two-dimensional distribution of reconstructed flame temperature under different ray numbers and different cases of *SNR*.

**Figure 12:**
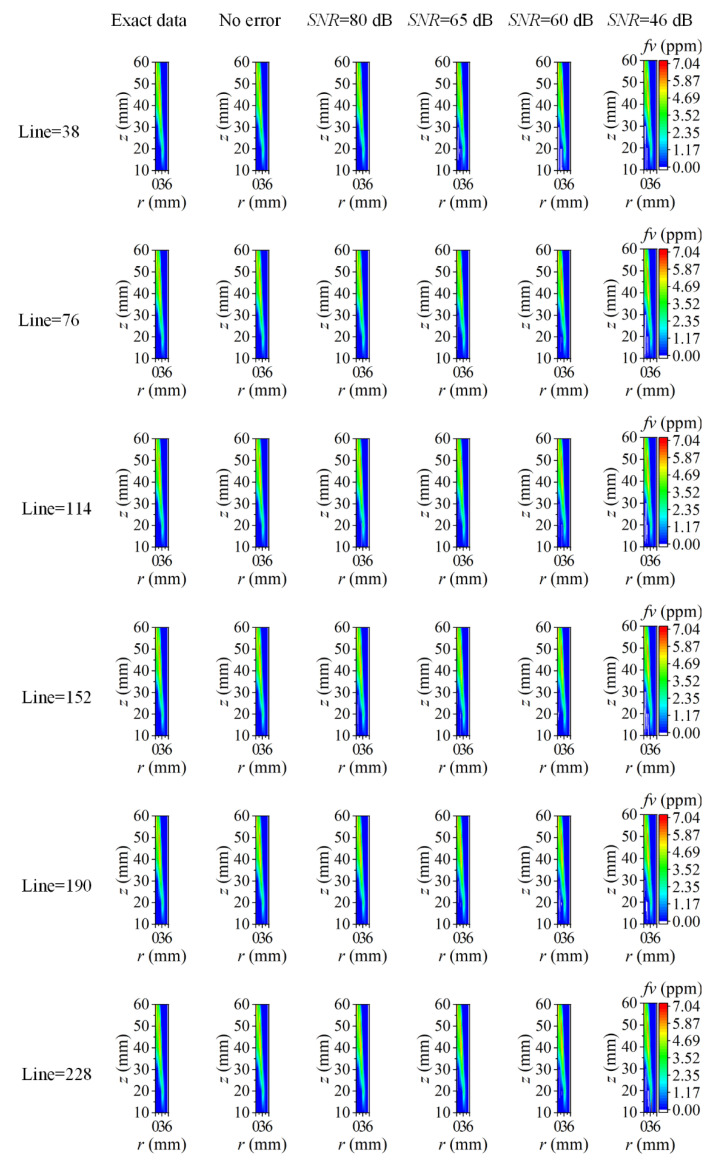
The two-dimensional distribution of reconstructed soot volume fraction under different ray numbers and different cases of *SNR*.

The reconstructed two-dimensional flame temperature and soot volume fraction using different ray numbers under different *SNR* are shown in Figure 11 and Figure 12, respectively. When there was no measurement error introduced in the radiation intensity, both the reconstructed flame temperature and the soot volume fraction were agreeable with the exact data. As the *SNR* of the measurement system decreased, as shown in Figure 11, there were many higher reconstructed temperature results near the central area. When the ray number was small, these higher flame temperatures occupied a large area, but as the ray number increased, the flame area with higher temperatures diminished and turned to the form of local small areas containing noise. At the same time, in addition to the higher-temperature area, there were also some parts of obvious lower temperature in the central regions. These areas with large reconstruction errors were mainly distributed within the sooting regions; nonetheless, the exact location was not fixed. As for the soot volume fraction, there were some negative reconstruction results within the sooting regions. These negative soot volume fractions appeared in the form of local noise in small regions under the condition of high *SNR*, but the area with a negative soot volume fraction became larger with the decrease of *SNR*. The increase in the ray number could improve the occurrence of these noise points under the condition of fewer ray numbers; however, such improvement was finite when the ray number further increased. The large reconstruction error in the central area was due to the low radiation signal caused by the low soot volume fraction on the one hand; on the other hand, the radiation signal in the central area transferred across the flame with a longer path, which meant greater energy extinction.

**Figure 13:**
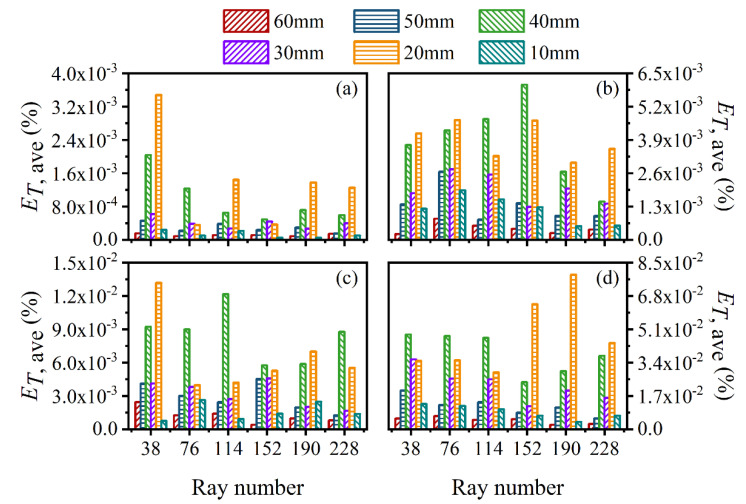
The average relative reconstruction error for the flame temperature at the main cross-sections in sooting regions. (**a**) *SNR* = 80 dB; (**b**) *SNR* = 65 dB; (**c**) *SNR* = 60 dB; (**d**) *SNR* = 46 dB.

**Figure 14:**
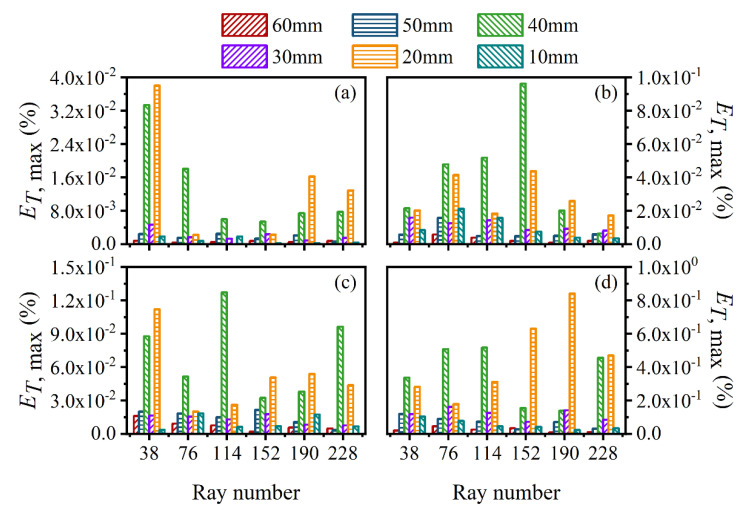
The maximum relative reconstruction error for the flame temperature at the main cross-sections in sooting regions. (**a**) *SNR* = 80 dB; (**b**) *SNR* = 65 dB; (**c**) *SNR* = 60 dB; (**d**) *SNR* = 46 dB.

**Figure 15:**
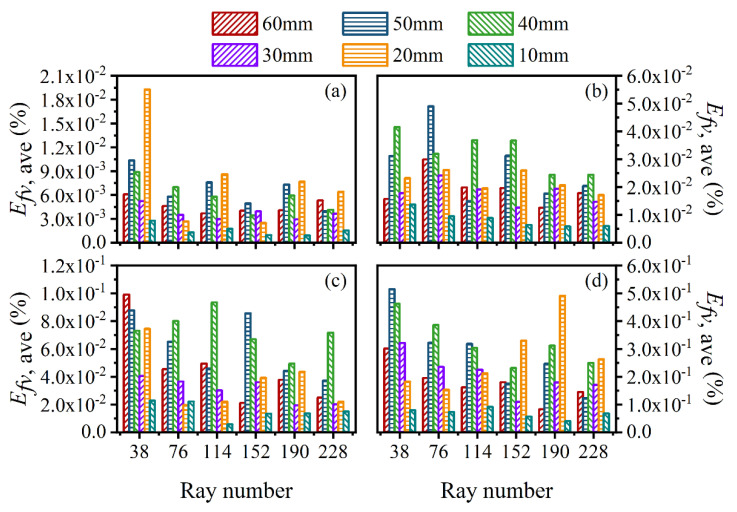
The average relative reconstruction error for soot volume fraction at the main cross-section in sooting regions. (**a**) *SNR* = 80 dB; (**b**) *SNR* = 65 dB; (**c**) *SNR* = 60 dB; (**d**) *SNR* = 46 dB.

**Figure 16:**
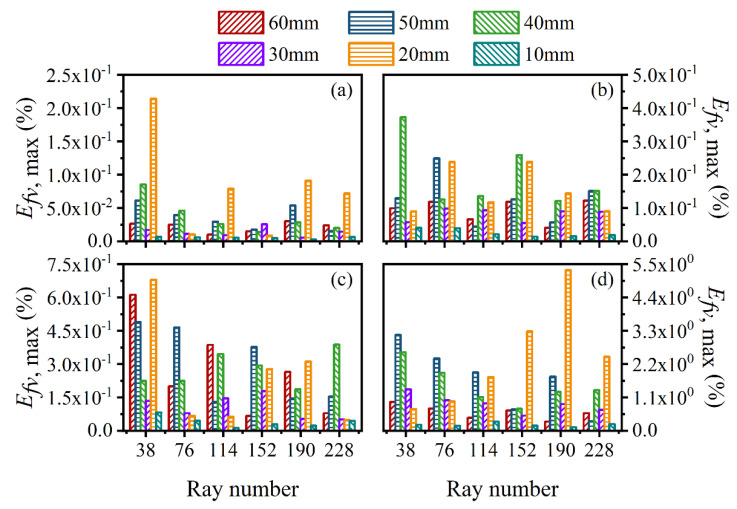
The maximum relative reconstruction error for soot volume fraction at the main cross-sections in sooting regions. (**a**) *SNR* = 80 dB; (**b**) *SNR* = 65 dB; (**c**) *SNR* = 60 dB; (**d**) *SNR* = 46 dB.

The two-dimensional reconstructed results in Figure 11 and Figure 12 reflect the reliability of the reconstruction accuracy on the whole, especially in sooting regions. To further verify the performance of the reconstruction procedure, the average and maximum relative reconstruction errors for both the flame temperature and soot volume fraction in the sooting regions are shown in Figure 13, Figure 14, Figure 15 and Figure 16. On the one hand, the relative reconstruction errors for the flame temperature and soot volume fraction were increased obviously with the decrease of the *SNR*; on the other hand, the improvement effect of the ray number on reconstruction accuracy became inconspicuous, especially because both the average and maximum relative reconstruction error of the temperature fluctuated with the increase of the ray number.

This was mainly because the grids of all the flame cross-sections were identical, and then the flame temperature and soot volume fraction were reconstructed using the same coefficient matrix, while the distributions of the temperature and soot concentration in each cross-section were different, which was equivalent to using the same spatial resolution to reveal the combustion characteristics of the flame cross-sections with different spatial distributions, and the reconstruction results might be distinct compared to the reconstruction for single flame cross-section. Meanwhile, when utilizing the image boundary as the unified reconstruction boundary, the meshing was aimed at the whole image area, which meant that the radiation rays crossed both the flame area and background area, hence, the effect of increasing the ray number on the reconstruction accuracy could affect the whole reconstruction area, while in the actual flame area, such influence became less apparent.

However, in general, the reconstruction accuracy of the flame temperature and soot volume fraction was high under different *SNR*, for example, when the *SNR* was as low as 46 dB, the maximum of the average and maximum relative reconstruction error for temperature was 0.078% and 0.84%, and the maximum of the average and maximum relative reconstruction error for soot volume fraction was 0.52% and 5.30%. In addition, the maximum relative reconstruction error of the soot volume fraction was mostly controlled by 5%.

**Figure 17:**
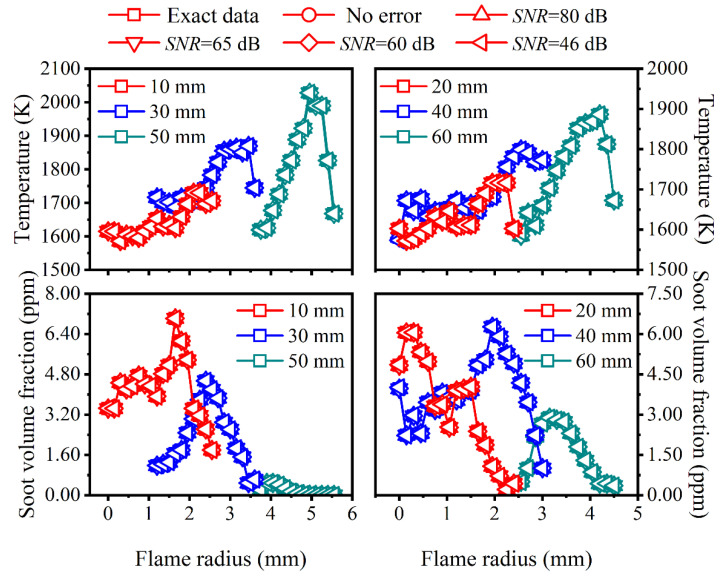
The reconstructed flame temperature and soot volume fraction at main cross-sections in the sooting regions using the ray number 228.

Although the positive correlation between the ray number and the reconstruction accuracy became less obvious, the relative reconstruction error of the flame temperature and soot volume fraction was lower using a higher ray number on the whole. Consequently, 228 was chosen as the optimal ray number for the subsequent calculation. The radial distributions of the temperature and soot volume fraction in the sooting regions are shown in Figure 17. The reconstructed flame temperature and soot volume fraction were in good agreement with the actual values under different *SNR* regardless of whether the flame temperature and soot concentration were continuously distributed from the center to the outside in the radial direction, which further illustrated the reliability of the reconstruction model.

In the previous discussion, the soot volume fraction in the non-sooting regions was assumed to be a fairly small value to ensure a continuous distribution of the soot volume fraction in the artificially divided flame elemental rings, which exhibited a large reconstruction error in the central area of the flame. To illustrate the influence of the soot volume fraction on the reconstruction performance, the soot volume fraction in the center of the non-sooting regions was supposed to be close to the value of the sooting regions, that was, the soot volume fraction distributed continuously along the radius and gradually decreased as a whole. The maximum radius of the flame (*r* = 5.55 mm) was utilized during the reconstruction, and the number of the flame elemental rings was 38.

**Figure 18:**
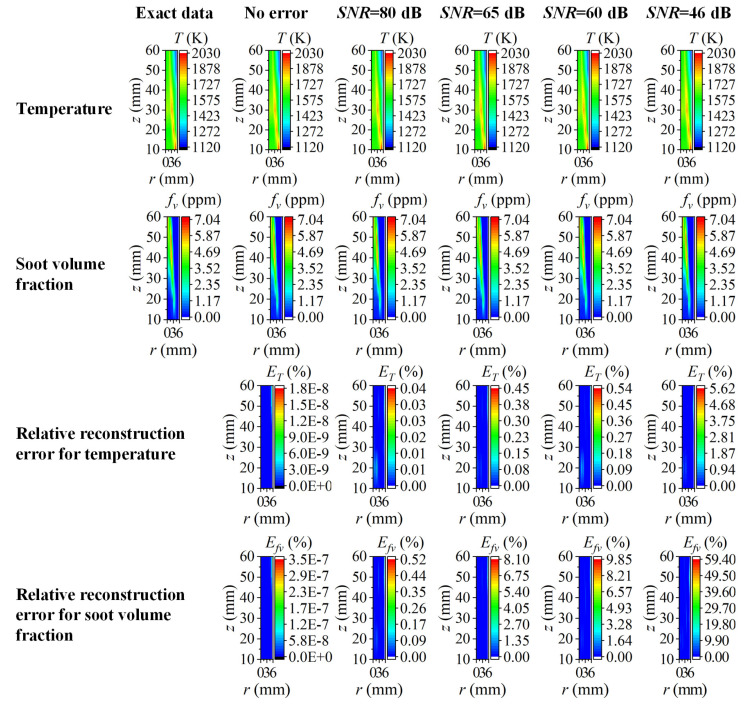
The reconstructed 2D flame temperature and soot volume fraction with corresponding relative reconstruction error at different *SNR*.

The reconstructed two-dimensional distribution of the flame temperature and soot volume fraction as well as the corresponding reconstruction error are displayed in Figure 18. The flame temperature and soot volume fraction were in good agreement with the assumed actual values, and there were no obvious higher or lower values in the flame temperature in the soot region, also there were no negative reconstructed values in the soot volume fraction either. According to the relative reconstruction error in the reconstruction area, nonetheless, the reconstruction results were still disturbed by the radiation intensity measurement error. The maximum relative reconstruction error of the flame temperature and soot volume fraction was 0.54% and 9.99% in the whole two-dimensional plane when the *SNR* was not lower than 60 dB. However, when the *SNR* was as low as 46 dB, the relative reconstruction error became unacceptable with the value of the temperature up to 5.6% and the value of the soot volume fraction up to 59%. It should be noted that the area with the large reconstruction error was merely concentrated in the central part of the flame and several locations of the reconstruction boundary, and the integral reconstruction accuracy was improved evidently when the values of the soot concentration in the central part of the flame were considerable.

**Figure 19:**
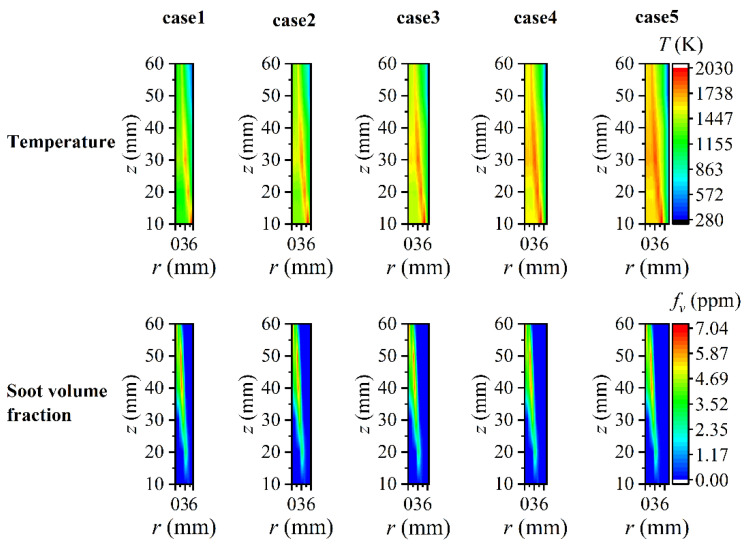
The exact flame temperature and soot volume fraction considering different reconstruction boundaries.

In the foregoing exposition, the maximum radius of the flame (*r* = 5.55 mm) was chosen as the reconstruction boundary, while the determination of the maximum radius of the flame from a practical flame image was also uncertain. Consequently, it was considered that the reconstruction boundary gradually extended to the outside of the flame to *r* = 6 mm, *r* = 6.45 mm, *r* = 6.90 mm, and *r* = 7.35 mm, respectively. These positions were denoted as case2, case3, case4, and case5, while the case of *r* = 5.55 mm was denoted as case1 to be a control group. The assumed flame temperature and soot volume fraction under five cases are shown in Figure 19. It should be pointed out that the flame temperature and soot volume fraction were identical within the same boundary range, while the only difference was that the flame temperature gradually decreased as the boundary expanded outward. During the reconstruction process, the number of flame elemental rings was divided with Δ*r* = 0.15 mm as the interval. The ray number 228 was still chosen as the optimal ray number for the reconstruction. Although, the optimal ray number needed to be redefined when the number of the flame elemental rings was different, the reconstruction accuracy was considerable when the ray number far outweighed the number of the flame elemental rings according to the preceding results. The maximum number of the flame elemental rings was 50 under five cases, so the ray number 228 was thought to be adequate for the reconstruction.

**Figure 20:**
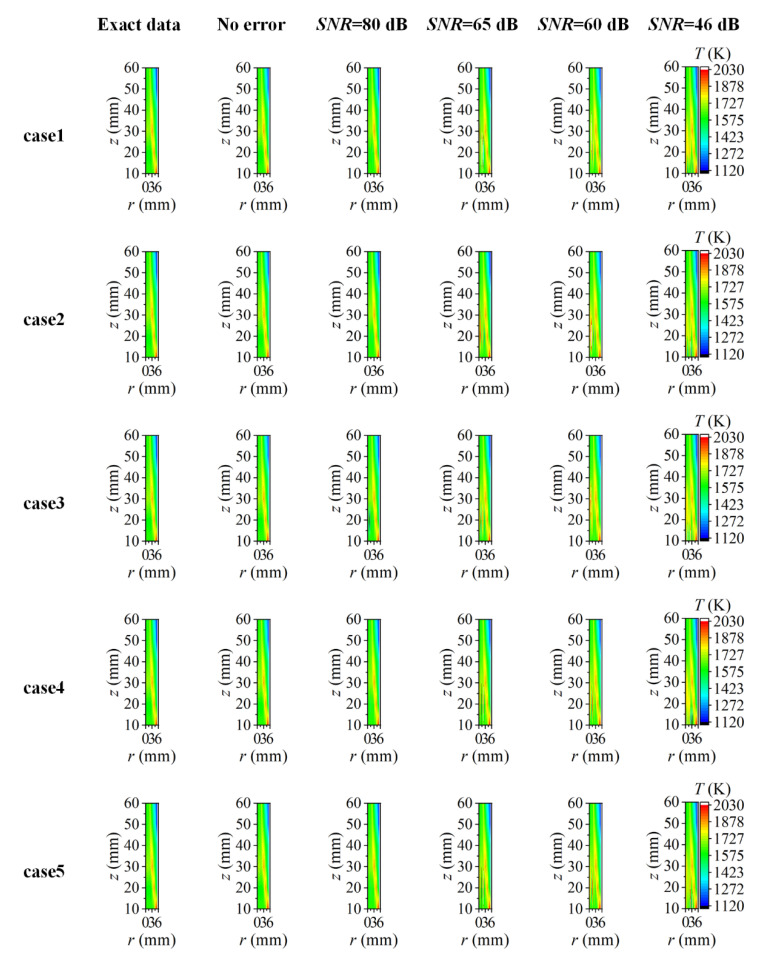
The reconstructed 2D flame temperature under different *SNR* considering different reconstruction boundaries.

**Figure 21:**
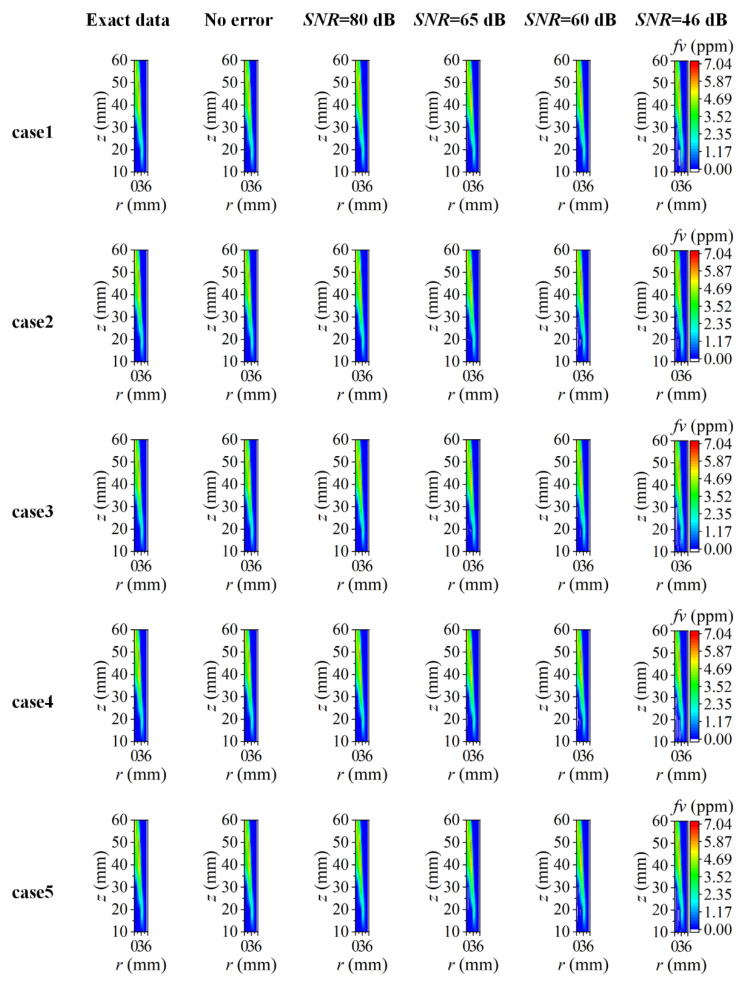
The reconstructed 2D soot volume fraction under different *SNR* considering different reconstruction boundaries.

The reconstructed flame temperature and soot volume fraction are displayed in Figure 20 and Figure 21, respectively. To facilitate comparison, the results presented in case2-case5 were all the reconstructed results captured at *r* = 5.55 mm to ensure one-to-one correspondence with the reconstructed position of case1. The flame temperature and soot volume fraction were reconstructed with high precision over the whole two-dimensional distribution under the condition of a high *SNR* of the system. As the *SNR* decreased gradually, the reconstruction performance in the central area of the flame became worse with a generally higher temperature and negative soot volume fraction. As the reconstruction boundary expanded outward, there were more values of a higher reconstructed temperature in non-sooting regions near the flame center, and there were also some lower reconstructed values when the *SNR* was as low as 46 dB. In comparison to the flame temperature, the soot volume fraction was less sensitive to the expansion of the reconstruction boundary. As the reconstruction boundary expanded, the difference in the soot volume fraction in the non-sooting regions close to the flame center was small under five cases, while the negative soot volume fraction appeared when the *SNR* was low. In addition, there was no significant correlation between the size and location of the negative soot volume fraction and the change of the reconstructed boundary. The difference in the influence of the reconstructed boundary on the temperature and soot concentration in the central area of the flame may be caused by the difference in their sensitivity to the measurement error of the radiation intensity.

On the one hand, the extension of the reconstruction boundary caused the introduction of more radiation intensity values with low signals in the reconstruction. On the other hand, the interval between the flame elemental rings was the same in five cases, and consequently, the numbers of the flame elemental rings increased with the expansion of the reconstruction boundary, which meant that the augmentation of the radiation source terms needed to be solved. Furthermore, the increased radiation source terms were different in their order of magnitude from those in the soot regions and the solution of them would be challenging especially under the condition of low *SNR*. As discussed above, the reconstruction accuracy was worse in the flame center and the reconstruction of the soot concentration was more sensitive to the measurement error. Therefore, the variation of the reconstruction boundary had a relatively significant influence on the reconstruction of the temperature in the non-sooting regions near the flame center, while for the soot volume fraction, the effect of the measurement error caused the appearance of the reconstruction values with large errors generally, and the impact of the reconstruction boundary became less intuitionistic. However, the flame temperature decreased by degrees and the soot concentration was extremely diminutive outside the maximum flame radius. Therefore, the radiation intensity introduced by the extension of the reconstruction boundary was very small compared to the radiation intensity of the flame in the sooting regions; there was little influence on the reconstruction of the flame temperature and soot volume fraction in the sooting regions as a consequence.

**Figure 22:**
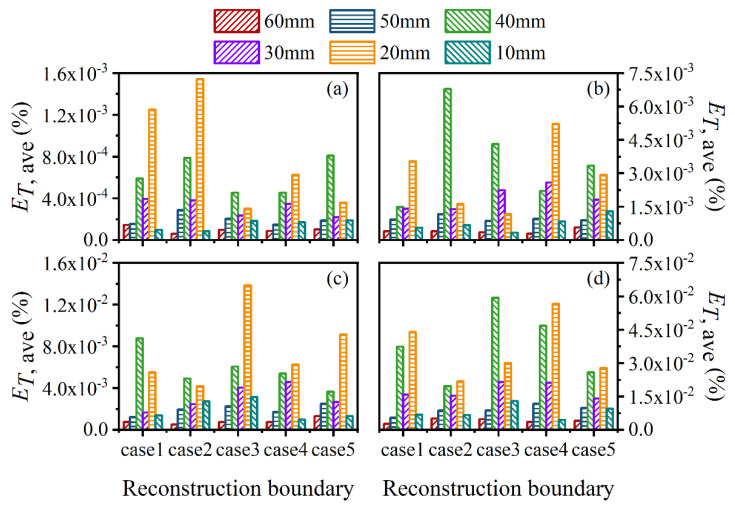
The average relative reconstruction error for temperature with different *SNR* considering different reconstruction boundaries. (**a**) *SNR* = 80 dB; (**b**) *SNR* = 65 dB; (**c**) *SNR* = 60 dB; (**d**) *SNR* = 46 dB.

**Figure 23:**
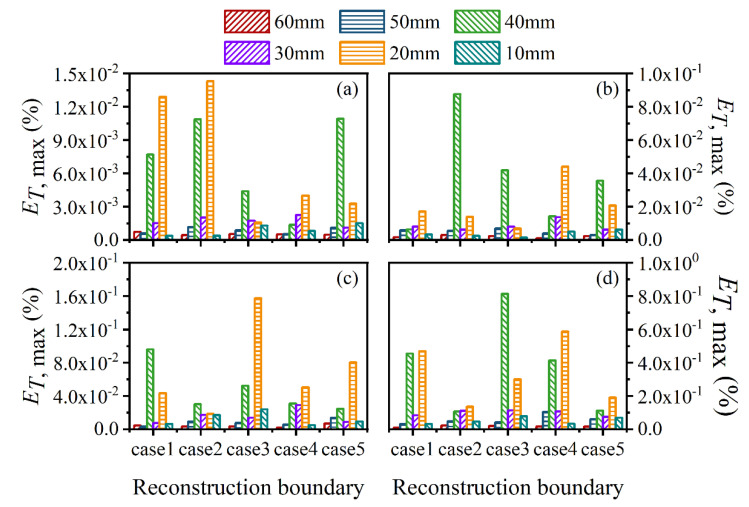
The maximum relative reconstruction error for temperature with different *SNR* considering different reconstruction boundaries. (**a**) *SNR* = 80 dB; (**b**) *SNR* = 65 dB; (**c**) *SNR* = 60 dB; (**d**) *SNR* = 46 dB.

**Figure 24:**
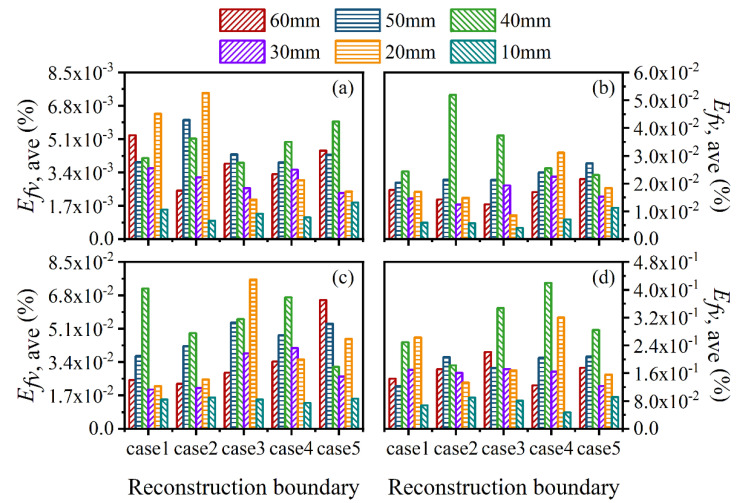
The average relative reconstruction error for soot volume fraction with different *SNR* considering different reconstruction boundaries. (**a**) *SNR* = 80 dB; (**b**) *SNR* = 65 dB; (**c**) *SNR* = 60 dB; (**d**) *SNR* = 46 dB.

**Figure 25:**
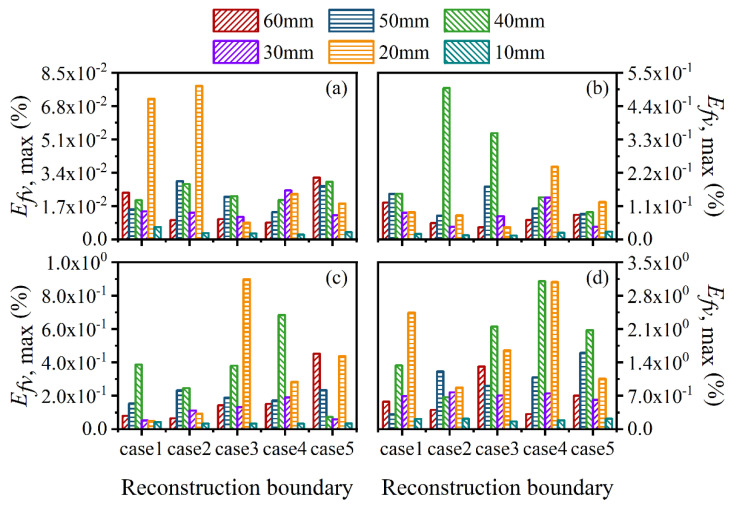
The maximum relative reconstruction error for soot volume fraction with different *SNR* considering different reconstruction boundaries. (**a**) *SNR* = 80 dB; (**b**) *SNR* = 65 dB; (**c**) *SNR* = 60 dB; (**d**) *SNR* = 46 dB.

For further verification of the above results, the average and maximum relative reconstruction errors of the flame temperature and soot volume fraction in the sooting regions were calculated, as shown in Figure 22, Figure 23, Figure 24 and Figure 25. It can be observed that the flame temperature and soot volume fraction in the sooting regions could be reconstructed with reliable accuracy in different reconstruction boundaries even when the *SNR* was low at 46 dB. The average and maximum relative reconstruction errors for the flame temperature were 0.06% and 0.81%, respectively, and the values for the soot volume fraction were 0.42% and 3.1%, respectively. Meanwhile, it was apparent that the relative reconstruction error of both temperature and soot volume fraction did not show obvious regularity under different reconstruction boundaries, which further indicated that the reconstructed boundary selected in the above range had no obvious influence on the reconstruction accuracy of the flame temperature and soot concentration in sooting regions.

## 4. Conclusions

In this paper, the influence of the edge effect on the reconstruction of the axisymmetric flame temperature and soot volume fraction by a single CCD camera was investigated in detail. The main conclusions are as follows.

1. The reconstruction accuracy of the flame temperature and soot volume fraction was insensitive to the measurement error of the coefficient matrix. The temperature and soot volume fraction could still be reconstructed reliably when the *SNR* of the measurement system for both the radiation intensity and coefficient matrix was as low as 46 dB;
2. The reconstruction of the flame temperature and soot volume fraction was greatly influenced by the edge effect. When the flame edge with weak radiation signals was ignored, the relative reconstruction error for the temperature and soot volume fraction increased from the flame center to the edge, and the soot volume fraction was influenced more obviously;
3. The flame image boundary could be chosen as the unified reconstruction boundary to reconstruct the two-dimensional distribution of the temperature and soot volume fraction with satisfactory accuracy. The low soot volume fraction could influence the reconstruction accuracy for both the temperature and soot concentration in non-sooting regions. These things considered, there was no obvious regularity between the reconstruction accuracy and the extension of the reconstruction boundary.

## Data Availability

Not applicable.
